# A Rare Case of Neonatal Escherichia coli Conjunctivitis With Maternal Asymptomatic Bacteriuria

**DOI:** 10.7759/cureus.92936

**Published:** 2025-09-22

**Authors:** Benjamin Merzouk, Kate Schwartzman, Panitan Yossuck, Vignesh Gunasekaran

**Affiliations:** 1 Pediatrics, West Virginia University School of Medicine, Martinsburg, USA; 2 Neonatology, West Virginia University School of Medicine, Martinsburg, USA

**Keywords:** asymptomatic bacteriuria, escherichia coli, neonatal conjunctivitis, neonatal infection, ophthalmia neonatorum, preterm infant

## Abstract

*Escherichia coli* is an uncommon but increasingly recognized pathogen in neonatal infections, including conjunctivitis. We report a case of a preterm male infant born at 34 weeks of gestation who developed *E. coli* conjunctivitis during his neonatal intensive care unit (NICU) stay. This case underscores the importance of considering *E. coli* as an emerging cause of neonatal conjunctivitis, particularly in preterm infants. It also highlights the critical role of thorough maternal history review, including recent infections, colonization, and obstetric complications, in guiding early diagnosis and targeted management. As antibiotic resistance patterns evolve and maternal colonization with Gram-negative organisms becomes more prevalent, clinicians must maintain a high index of suspicion for atypical pathogens in neonatal infections.

## Introduction

Neonatal conjunctivitis (ophthalmia neonatorum) affects 1%-2% of live births in high-income countries, predominantly within the first month of life, and is historically linked to the perinatal transmission of *Neisseria gonorrhoeae* and *Chlamydia trachomatis* [1]. Universal prophylaxis with 0.5% erythromycin ointment has significantly decreased gonococcal disease; however, it has a limited effect on chlamydia and a negligible impact on other bacterial species. Contemporary surveillance reveals a rising proportion of conjunctivitis due to enteric Gram-negative rods - *Escherichia coli*, *Klebsiella*, *Pseudomonas *- particularly in intensive care settings [2]. Despite their rarity, these organisms pose significant risks as frequent causes of neonatal sepsis that may lack standardized treatment algorithms.

We describe a culture-confirmed *E. coli* ophthalmia neonatorum in a late-preterm infant with maternal intrapartum bacteriuria, situating the case within the existing literature and current management recommendations. The report adheres to the Consensus-based Standards for the Reporting of Clinical Cases (CARE) checklist to optimize transparency and educational value.

## Case presentation

Maternal course

A 27-year-old Caucasian G4P0212 with a significant antenatal history of intrahepatic cholestasis was treated with ursodiol. She had previous pre-eclampsia and at 33-4/7 weeks, she presented with a blood pressure of 160/95 mmHg, headache, blurry vision, and elevated hepatic transaminases. The SARS-CoV-2 infection diagnosed two weeks earlier, at 31-6/7 weeks, was mild (presented with complaints of headache, pruritus). Urine analysis showed moderate leukocytes when she presented with complaints of dark colored urine; subsequent urine culture grew to >20,000 CFU/mL *E. coli* but deemed asymptomatic bacteriuria. Other prenatal labs, such as Hepatitis B, human immunodeficiency virus (HIV), and rapid plasma reagin (RPR), were negative. No history of herpes simplex virus (HSV) infection was documented.

Intrapartum course

Induced vaginal birth at 34-0/7 weeks after two betamethasone doses and adequate intrapartum penicillin coverage for positive Group B *Streptococcus* (GBS) status. The duration of rupture of membranes to delivery was two hours, and there were no signs of maternal chorioamnionitis. The male infant, delivered vaginally, was on room air (RA) at birth and was placed skin to skin with the mother for 20 minutes. Birth weight was 2.22 kg (47th percentile). The infant received Vitamin K, erythromycin ocular prophylaxis, and hepatitis B vaccine at birth.

Clinical course

The infant was admitted to the neonatal intensive care unit (NICU) for prematurity. Initially stable on RA, he developed hypoxia requiring continuous positive airway pressure (CPAP) of 5 cm H₂O and an FiO_2_ requirement of 30%. Chest radiography reported mild haziness consistent with respiratory distress syndrome (RDS). He received one dose of surfactant via the less invasive surfactant administration (LISA) technique and was started on caffeine for apnea of prematurity. Due to clinical illness with increasing oxygen requirements, a limited sepsis evaluation was performed. With ongoing respiratory support, the infant was treated empirically with ampicillin and gentamicin for five days despite a negative blood culture.

**Figure 1:**
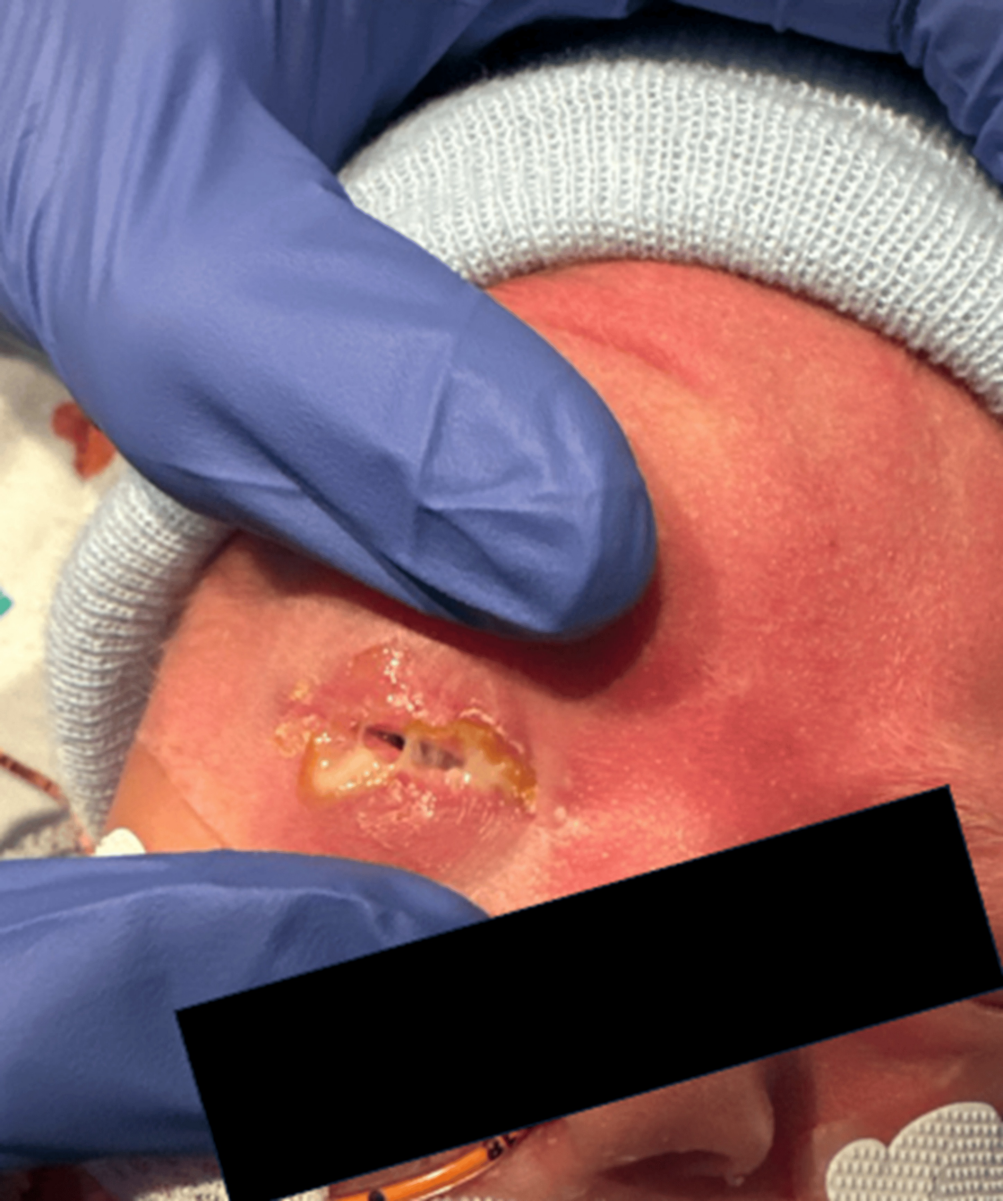
Purulent discharge of the right eye in a preterm newborn with Escherichia coli conjunctivitis

On day of life (DOL) 7, the infant developed right eyelid swelling with profuse purulent discharge (Figure 1). An examination revealed palpebral conjunctival erythema in the right eye, with a clear bulbar conjunctiva. Discharge was sent for bacterial culture and nucleic acid amplification testing (NAAT) for *N. gonorrhoeae* and *C. trachomatis*. Laboratory evaluation showed a normal white blood cell count (13.5×10³/µL), normal band count, and C-reactive protein (CRP) of 1.1 mg/L. Blood cultures were repeated, and Pediatric Infectious Disease was consulted. Empiric treatment included a three-day course of IV azithromycin, a single dose of IV ceftriaxone (to treat possible gonococcal infection), and topical antibiotics (ofloxacin and erythromycin) administered to both eyes every six hours.

Eye culture grew gram-negative rods, later identified as *E. coli*. A lumbar puncture was performed, and empiric antimicrobial therapy was initiated with ampicillin, ceftazidime, and acyclovir. Cerebrospinal fluid (CSF) analysis revealed: protein 114 mg/dL, glucose 51 mg/dL, red blood cells 6/µL, and nucleated cells 15/µL. The herpes simplex virus polymerase chain reaction (HSV PCR), blood, and cerebrospinal fluid (CSF) cultures were negative. Acyclovir was discontinued following negative HSV results. With negative cultures, ampicillin was discontinued, but ceftazidime was continued based on *E. coli* susceptibility. After five days of intravenous antibiotics, the infant was transitioned to amoxicillin-sulbactam and completed a 10-day antibiotic course. Topical antibiotics were discontinued on day 7 of illness as ocular symptoms resolved. Both eyes appeared normal with no signs of conjunctivitis.

The infant was weaned to RA by DOL 10. Enteral feeds were gradually advanced via nasogastric tube, and he transitioned to full oral feeds by three weeks of life. Ophthalmologic evaluation revealed no anterior or posterior segment pathology. The infant was discharged home on DOL 28 with an outpatient pediatric follow-up. The sequence of events is listed in Table 1.

**Table 1:** Timeline of the Clinical Course

Day (chronologic)	Maternal / Infant Events	Interventions
-14 (pre-delivery)	Maternal asymptomatic *Escherichia coli* bacteriuria 20,000 CFU/mL; mild COVID-19 infection	None
-2 (pre-delivery)	Maternal admission for intrahepatic cholestasis of pregnancy	Betamethasone 6 mg, ursodiol
-1 (pre-delivery)	Maternal group B *Streptococcus* (GBS) prophylaxis	Penicillin 5 million units
0 (Delivery)	Vaginal birth at 34-0⁄7 weeks; respiratory distress syndrome symptoms shortly after birth	Nasal continuous positive airway pressure (CPAP) 5 cm H₂O; fraction of inspired oxygen (FiO₂) 0.3
0-2	C-reactive protein (CRP) mildly elevated	Empiric ampicillin and gentamicin for limited sepsis, 5 days, surfactant via less invasive surfactant administration (LISA)
3-6	Gradual respiratory improvement	Weaned to FiO₂ 0.25, caffeine started
7	Right-eye swelling, purulent discharge	Eye Gram stain, cultures, and *Gonorrhea/Chlamydia* (GC/CT) nucleic acid amplification test (NAAT) sent; started azithromycin ×3 days, single ceftriaxone 100 mg/kg, topical erythromycin+ofloxacin
8	Eye culture preliminary: Gram-negative rods; CRP, 1.1 mg/L; complete blood count (CBC): white blood cells (WBC), 13.5 ×10⁹/L	Lumbar puncture: Protein 114 mg/dL, glucose 51 mg/dL, 15 WBC/µL; started ampicillin, ceftazidime, acyclovir
9	Final eye culture: *E. coli* sensitive to cefepime, ceftazidime, ampicillin-sulbactam, ceftriaxone; cerebrospinal fluid (CSF) culture negative; blood culture negative, herpes simplex virus polymerase chain reaction (HSV PCR) negative	Discontinued ampicillin and acyclovir; continued ceftazidime
10	On room air, eye discharge is decreasing	
11		Difficulty in obtaining intravenous (IV) access, ceftazidime discontinued, and switched to amoxicillin-sulbactam
14	Completed 10-day systemic antibiotics; topical therapy stopped on day 7 of illness	
21	Full oral feeds; ophthalmology exam normal anterior & posterior segments	
28	Discharged home; normal weight gain	Follow-up arranged with primary care

## Discussion

*E. coli* is known as a dangerous cause of maternal and neonatal sepsis resulting from colonization of the maternal gastrointestinal tract, spreading vertically to the fetus [3]. While the perinatal and postnatal transmission of *E. coli* is well-established, its intrauterine transmission remains unclear. Vertical transmission of disease during birth is a well-known phenomenon; however, the mechanism of *E. coli* transmission during birth is not well studied. This fact can be attributed, in part, to the elaborate virulence transposition between different pathovars of *E. coli* [4].

Maternal *E. coli* colonization demonstrates significant transmission potential to neonates. Studies reveal maternal colonization rates of 19.9% with vertical transmission occurring in 21.4% of cases [3]. Among very-low-birth-weight infants born to colonized mothers, transmission rates reach 26%, with *E. coli* emerging as the most frequently transmitted pathogen (35.6%). When *E. coli* is isolated from maternal blood, transmission rates to neonates reach 100%, with 33.3% of these cases developing neonatal sepsis. Additionally, *E. coli* demonstrates the highest transmission rate of 30.2% from the maternal vagina, with 15.1% of these cases leading to early-onset sepsis. Of the 10 cases of early onset, culture-proven sepsis in this study, eight were caused by *E. coli* [5].

The ascending infection pathway appears particularly relevant in preterm deliveries. Maternal colonization predisposes to ascending infection and placental involvement, with a significant correlation observed between maternal *E. coli* colonization and histopathologic placental inflammation [6]. Extended-spectrum beta-lactamase-producing *E. coli* (ESBL-E) demonstrates even higher risk, with 17.5% maternal colonization rates in threatened preterm labor and 50% vertical transmission rates. Most concerning, extended-spectrum beta-lactamase-producing *Enterobacterales* (ESBL-E)-colonized infants are delivered at earlier gestational ages and experience more complications [6].

Clinical presentation and differential diagnosis

Neonatal *E. coli* conjunctivitis presents with conjunctival injection and purulent discharge, making it difficult to distinguish clinically from the far more common causes of neonatal conjunctivitis: *N. gonorrhoeae* and *C. trachomatis* [7]. These conditions so often cause neonatal conjunctivitis that screening and treatment are mandated in World Health Organization standard-of-care guidelines [8]. However, despite the prevalence of *N. gonorrhoeae* and *C. trachomatis* in the NICU, clinicians should be cautious in generalizing neonatal eye infections to these two pathogens.

In a study of 65 NICU infants, 38% were found to have conjunctivitis caused by Gram-negative bacteria, the most prevalent of which was *Klebsiella*. *E. coli* accounted for 17% of Gram-negative conjunctivitis cases in NICU settings [2]. Contemporary surveillance reveals that Gram-negative bacteria cause 66% of healthcare-associated conjunctivitis in neonates, with *E. coli* representing 11.5% of all cases [9]. Critically, these bacteria are often not consistently covered by the macrolide regimen, indicating that blanket coverage may leave some neonates at risk [2].

The clinical features of *E. coli* ophthalmia neonatorum include normothermia, purulent discharge, and lid edema. Low birth weight (<1500 g) and low gestational age (delivery at or before 29 weeks) significantly increase suspicion for Gram-negative causes in NICU infants with clinical signs of conjunctivitis. Birth weight less than 1500 g (odds ratio (OR) 4.35) and gestational age of 29 weeks or fewer (OR 5.60) represent independent risk factors for gram-negative conjunctivitis development [2].

Asymptomatic bacteriuria: clinical significance and treatment controversies

The guidelines from the American College of Obstetricians and Gynecologists (ACOG), the Infectious Diseases Society of America (IDSA), and the US Preventive Services Task Force (USPSTF) align closely regarding asymptomatic bacteriuria (ASB) screening. All recommend screening for ASB during pregnancy with a midstream urine specimen and treating urine cultures that yield ≥100,000 CFU/mL with a multi-day course of antibiotics [10-12]. ACOG and IDSA further recommend screening only once at the start of pregnancy in the first trimester. While the USPSTF and ACOG acknowledge a changing epidemiologic landscape of pyelonephritis in pregnancy and high risk of bias in much of the original evidence recommending screening, the IDSA maintains that the size and consistency of benefit found in earlier research still strongly support screening for and treating ASB in pregnancy.

A 2023 systematic review of research since 2005 identified only four studies assessing birth outcomes in untreated ASB, likely due to the prevailing assumption that untreated ASB causes complications. Studies, therefore, focus on complications after treating ASB compared to women without ASB. The review reported untreated ASB strongly increased odds of pyelonephritis (n=4283; OR 3.9, 95% confidence interval (CI) (1.4-11.4), p<0.001) and modestly elevated risk of low-birth weight (n=212; RR 1.84, 95% CI (1.22-2.78), p=0.002) compared to women without ASB [13]. When treated with antibiotics, various studies reported the occurrence of pyelonephritis and low birth weight comparable to women without ASB. However, studies were not congruent on the impact of ASB on preterm birth, and 11 of the 13 studies were at risk for bias [13]. No studies were found that examined the relationship between asymptomatic bacteriuria caused by *E. coli* or GBS and the clinical outcomes in these infants.

GBS and subclinical bacteriuria

In a retrospective cohort study (n=305) from 2007, the presence of untreated group B streptococcal (GBS) asymptomatic bacteriuria with any colony count was associated with a 7.2-fold increase in adjusted odds of developing chorioamnionitis compared to uninfected women (95% CI: 2.4 to 21.2, p<0.05). The authors offered no treatment recommendation by CFU/mL due to the inclusion of three cases of untreated clinical bacteriuria (>100,000 CFU/mL), concluding these cases biased the cohort toward increased chorioamnionitis risk [14]. However, the strong association (adjusted odds ratio (aOR)=7.2) and high proportion of untreated subclinical bacteriuria (<100,000 CFU/mL) in the cohort (95%, 58/61) warrant concern for serious morbidity associated with subclinical bacteriuria.

Although the virulence of GBS differs from that of *E. coli*, this study and our case suggest significant morbidity may be associated with subclinical bacteriuria being missed in the literature. This may already be apparent to clinicians: 61% (27/44) of patients with bacteriuria >10,000 and <100,000 CFU/mL were treated in the cohort, indicating clinicians employ clinical judgment to mitigate sequelae from subclinical ASB.

Implications for clinical practice

This case provides an example of *E. coli* neonatal conjunctivitis transferred vertically, causing conjunctivitis shortly after birth. The presentation was unexpected because the mother had low-count *E. coli* bacteriuria, defined as insignificant by standard criteria. Along with illustrating this rare cause of a common neonatal concern, the case raises the question: Should mothers with bacteriuria be treated as at risk for vertical transmission, even if the count is low?

Regardless of guidelines in place, it is critical that neonatologists assess the maternal history carefully for unanticipated causes if a neonate presents with signs of vertically transmitted conjunctivitis. The high transmission rates of *E. coli* from maternal colonization, particularly in preterm infants, support enhanced surveillance and consideration of broader antimicrobial coverage when Gram-negative organisms are suspected. In our case, the limitations include a lack of molecular typing to link the maternal bacteriuria isolate with ocular strain.

## Conclusions

Enteric Gram-negative organisms account for up to 53% to 66% of culture-positive neonatal conjunctivitis. *E. coli* remains rare but is reported to be increasing, largely in preterm or low-birth-weight infants exposed to maternal genitourinary colonization. As described in prior cases in the literature, our patient was afebrile and systemically stable, but we opted for a full sepsis work-up given prematurity and potential for invasive disease. Current US guidelines emphasize systemic third-generation cephalosporin for suspected *N. gonorrhoeae* ophthalmia neonatorum and oral macrolide for chlamydia, with limited guidance for other pathogens. Some authors endorse topical monotherapy for well infants once sensitivities return. We continued systemic antibiotics because *E. coli* is a common cause of late-onset sepsis and meningitis

*Message to the clinician: *(a)* E. coli* should enter the differential for purulent neonatal conjunctivitis, particularly when Gram-negative rods are visualized; (b) premature infants warrant systemic evaluation and antibiotic therapy until invasive infection is excluded; (c) culture-guided de-escalation and early cessation of topical therapy minimize antimicrobial exposure without compromising outcomes.
